# A High Level of Fibrinogen Degradation Product on Arrival as the Only Clue Suggesting Deterioration in a Blunt Trauma Patient

**DOI:** 10.7759/cureus.30914

**Published:** 2022-10-31

**Authors:** Youichi Yanagawa, Hiroki Nagasawa, Kouhei Ishikawa

**Affiliations:** 1 Acute Critical Care Medicine, Juntendo University Shizuoka Hospital, Izunokuni, JPN

**Keywords:** fdp, cardiac arrest, hyperkalemia, hemorrhage, blunt trauma

## Abstract

We report the case of an 89-year-old woman who was struck by a car while walking and fell to the ground. She had hypertension, dyslipidemia, and cerebral infarction requiring medication. She was transported to a nearby acute critical care center. Upon arrival, her vital signs were stable. A physical examination showed right facial and hip contusion, right shoulder tenderness, a right elbow contusional lacerated wound, and bilateral knee abrasion wounds. She vomited when her face moved. Radiological studies showed a right proximal humerus fracture and a right minor ischial fracture. Her injury severity score (ISS) was 5 points, and her probability of surviving was 97.8%. However, a blood test revealed an extremely high fibrinogen degradation product (FDP) level (573.3 μg/mL). Because of this elevated FDP value and her inability to walk due to vomiting on motion, she remained in the emergency room (ER) for monitoring. At five hours from arrival, she became comatose, and hypotension and bradycardia (30 beats per minute) were noted followed by cardiac arrest. She underwent advanced cardiac life support and obtained spontaneous circulation. Repeated blood tests showed hyperkalemia, anemia, and hypoglycemia. She immediately underwent infusion of glucose and insulin and continuous infusion of catecholamine. Repeated whole-body CT scans revealed only increased hematomas where the fractures and contusions existed. She was admitted to the ICU. Her post-admission course was quite eventful. She required transfusion until the fourth hospital day to control circulation and anemia and underwent transfusion of 28 units of red blood cells, 30 units of platelets, and four units of fresh-frozen plasma in total. After her circulation and respiratory function had stabilized, she was extubated. However, her condition became complicated with the deterioration of her knee wounds and gall bladder inflammation in the ward. All complications were treated by non-operative management. She was transferred to another hospital for rehabilitation on day 70.

This report discusses our experience with a blunt trauma patient in whom a high FDP level on arrival was the only clue indicating the deterioration of her condition. Such patients need close observation with hospitalization.

## Introduction

The major cause of unstable circulation after trauma in the acute phase is usually hemorrhage followed by neurogenic issues induced by spinal cord injury and/or obstruction induced by tension pneumothorax or cardiac tamponade [1,2]. However, in rare cases, preceding endogenous diseases can cause shock in trauma patients [3]. We report the case of a blunt trauma patient in whom a high fibrinogen degradation product (FDP) level on arrival was the only clue indicating the deterioration of her condition. Despite her vital signs otherwise being stable and her condition of moderate severity on arrival, the patient suffered cardiac arrest while awaiting admission.

## Case presentation

The patient was an 89-year-old woman who was struck by a car while walking and fell to the ground. She had hypertension, dyslipidemia, and cerebral infarction treated by azilsartan/amlodipine, rosuvastatin, clopidogrel, etizolam, and epinastine. She was transported to a nearby acute critical care center.

**Figure 1:**
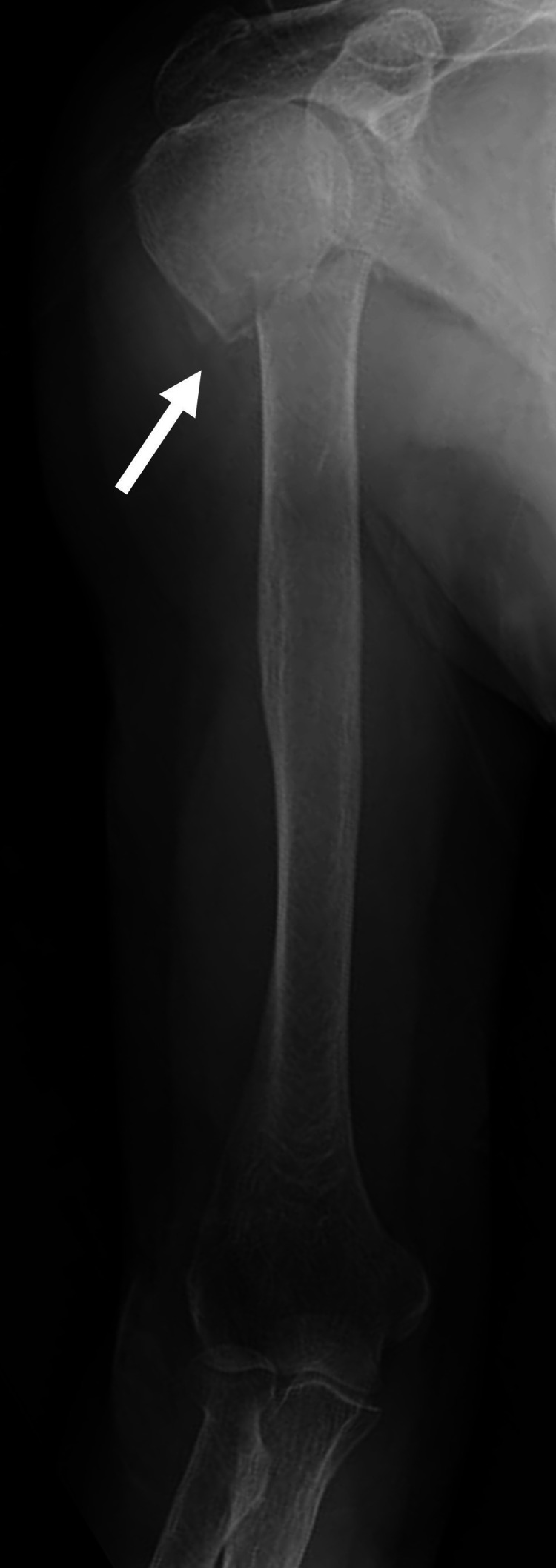
X-ray study of the humerus on arrival X-ray showed a right proximal fracture (arrow)

**Figure 2:**
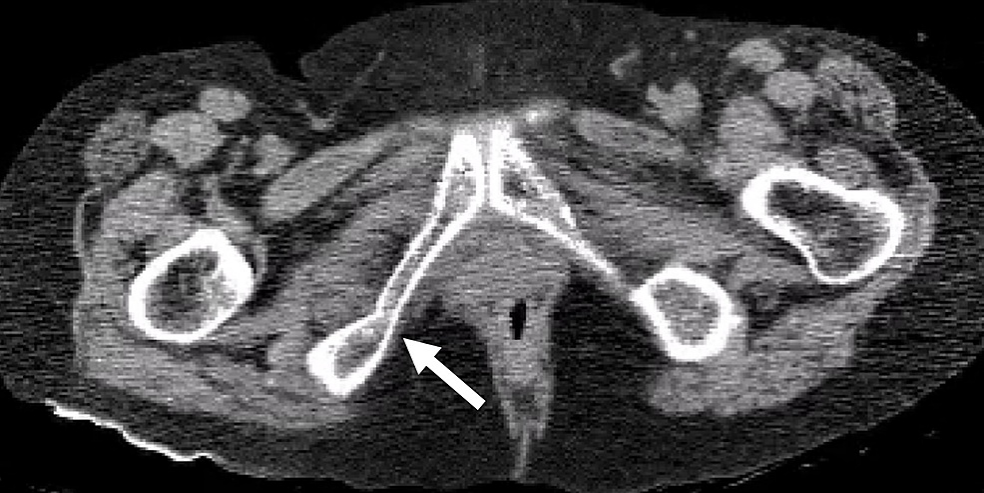
CT findings on arrival CT showed a right minor ischial fracture (arrow) CT: computed tomography

Upon arrival, her vital signs were as follows: clear consciousness; blood pressure of 131/76 mmHg; heart rate of 71 beats per minute; respiratory rate of 22 breaths per minute; percutaneous saturation of 99% under 3 L/minute of oxygen via mask; and body temperature of 36.9 °C. A physical examination showed right facial and hip contusion [Abbreviated Injury Scale (AIS) score = 1, respectively], right shoulder tenderness, right elbow contusional lacerated wound (AIS = 1), and bilateral knee abrasion wounds (AIS = 1). She vomited when her face moved. She did not show any sign of spinal cord injuries. The results of a venous blood gas analysis were as follows: pH: 7.424; PCO_2_: 39.1 mmHg; PO_2_: 52.6 mmH; HCO_3_-: 25.0 mmol/L; base excess: 0.3 mmol/L, and lactate: 1.25 mmol/L. Radiological studies showed a right proximal humerus fracture and a right minor ischial fracture (Figures 1, 2).

Her injury severity score (ISS), which was calculated as the sum of the squares of the highest AIS code in each of the three most severely injured ISS body regions (head or neck, face, chest, abdomen, extremities or pelvis, and external), was 5 points [(face = 1^2^) + (extremities or pelvis = 2^2^)], revised trauma score was 7.8408, and probability of surviving was 97.8%. The main blood test findings were as follows: leukocytosis (10,600/μL), anemia (hemoglobin of 10.7 g/dL), and a high level of FDP (573.3 μg/mL) (Table 1).

**Table 1:** Results of the blood test eGFR: estimated glomerular filtration rate

Variables	On arrival	In 6 hours	Second day	Normal range	Unit
White blood cell count	10,600	24,500	16,100	3,600 – 8,900	/μL
Hemoglobin	10.7	6.7	8.7	11.1 – 15.2	g/dL
Platelet	23.3 × 10^4^	14.7 × 10^4^	8.7 × 10^4^	15.3 – 34.6 × 10^4^	/μL
Total protein	6.6	4.6	4.8	6.5 – 8.5	g/dL
Aspartate aminotransferase	39	43	4,770	5 – 37	U/L
Alanine aminotransferase	35	33	3,834	6 – 43	U/L
Lactate dehydrogenase	505	571	6,935	124 – 222	U/L
Creatinine phosphokinase	73	70	315	47 – 200	U/L
Amylase	63	104	808	43 – 124	U/L
Blood urea nitrogen	11.9	15.9	29.5	9 – 21	mg/dL
Creatinine	0.6	1.08	1.54	0.5 – 0.8	mg/dL
eGFR	69	36	25	70	mL/min
Sodium	143	142	143	135 – 145	mmol/L
Potassium	4.3	7.4	3.6	3.5 – 5.0	mmol/L
Chloride	107	109	105	96 – 107	mmol/L
Calcium	8.7	8.3	7	8.8 – 10.6	mg/dL
C-reactive protein	0.07	0.09	2.96	Under 0.30	mg/dL
Glucose	123	49	90	65 – 109	mg/dL
Fibrinogen degradation products	573.3	406.8	304.9	Under 10	μg/ml
Activated partial thromboplastin time	26.8	42.8	28.2	30	Seconds
Prothrombin time international normalized ratio	0.96	1.81	1.52	1 ± 0.1	

Because of the patient's elevated FDP value and her inability to walk due to vomiting on motion, she remained in the emergency room (ER) for monitoring after her wounds had been sutured until she could move to a ward. The humerus fracture was treated by a fixed sling. While waiting to be moved, she developed temporary hypotension with bradycardia that was corrected by Ringer's lactate solution. The urine output was not measured. Her contusional area became swollen, and she became drowsy. At five hours from arrival, she became comatose in addition to having worsening hypotension and bradycardia (30 beats per minute). The infusion of 0.25 mg of adrenalin was not effective, and she entered cardiac arrest.

She underwent chest compression, tracheal intubation with mechanical ventilation, and an additional infusion of 1 mg of adrenalin. After two minutes, she obtained spontaneous circulation. An arterial blood gas (FiO_2_ of 1.0) analysis showed the following values: pH: 7.237; PCO_2_: 22.6 mmHg; PO_2_: 368.4 mmH; HCO_3_-: 9.4 mmol/L; base excess: 18.0 mmol/L; lactate: 12.5 mmol/L; glucose: 49 mg/dL; potassium: 7.4 mEq/L; and hemoglobin: 6.7 g/dL. She immediately underwent infusion of glucose, insulin transfusion, and continuous infusion of catecholamine. Repeated whole-body CT revealed only increased hematomas where the fractures and contusions existed.

She was admitted to the ICU. Her post-admission course was very eventful. On the second hospital day, her condition was complicated by shock liver, renal failure, hyperamylasemia, and heart failure (Table 1). She required transfusion until the fourth hospital day to control circulation and anemia and underwent transfusion of 28 units of red blood cells, 30 units of platelets, and four units of fresh-frozen plasma in total. After her circulation and respiratory function had stabilized, she was extubated. She was transferred to the orthopedic ward on day eight. Her condition again became complicated with the deterioration of her knee wounds and gall bladder inflammation in the ward. All complications were treated by non-operative management. She was transferred to another hospital for rehabilitation on day 70.

## Discussion

Our patient had stable vital signs and only moderately severe trauma on the AIS but later developed cardiac arrest. The high FDP level on arrival was the only clue suggesting the deterioration of the condition in this blunt trauma patient. The cardiac arrest was due to concealed hemorrhaging at the contusional and fracture sites that was accelerated by platelet aggregation inhibitor as well as hyperkalemia accelerated by renal dysfunction. The hyperkalemia was considered to be the main cause of the cardiac arrest in this patient because she had not shown any tachycardia in the ER.

The ISS of the present case was 5 points, and her probability of surviving was 97.8%, and hence the physiological and anatomical severity on arrival failed to predict cardiac arrest during hospitalization. However, the patient showed a markedly increased level of FDP, a biomarker used to predict the outcome in trauma patients [4-6]. Even when vital signs are stable and trauma is not very severe, a high FDP level on arrival may predict the need for transfusion or a poor outcome [4-6]. An FDP value exceeding 150 μg/ml on arrival suggests a potentially fatal outcome, so careful observation should be undertaken for such patients [6]. Because FDPs are fragments released following plasmin-mediated degradation of fibrinogen or fibrin, the FDP level is very sensitive to intravascular thrombus and may be markedly elevated in cases of disseminated intravascular coagulation (DIC), acute aortic dissection, and pulmonary embolism [7]. It is possible that the patient's coagulation function had also deteriorated due to the trauma, and although no decrease in platelets was noted, it is important to be mindful of changes in the coagulation function [8]. The patient was waiting in the ER for monitoring, and thus she was able to receive immediate suitable resuscitation on collapse and survived after the administration of intensive care.

The main cause of cardiac arrest in the present case was hyperkalemia, in addition to hemorrhagic shock induced by hematomas developing at all injured sites. The hyperkalemia was thought to have been induced by potassium released from injured tissue, occult adrenal insufficiency due to aging that was manifested under severe stress conditions, occult kidney dysfunction due to aging, or the side effects of drugs [9-13]. Toxic substances, such as myoglobulin from injured muscle tissue, or unstable circulation due to hemorrhaging can also contribute to the deterioration of kidney function, resulting in hyperkalemia [14]. In hindsight, we should have conducted a blood gas analysis every few hours, which would have indicated the levels of hemoglobin and potassium, and so we could have noticed when the patient developed temporary hypotension with bradycardia. The major risk factor for hyperkalemia in the present patient was thought to be aging. However, even middle-aged individuals may sometimes die due to diffuse soft tissue injuries that occur after an assault [15]. Accordingly, older patients with simple contusions but high levels of FDP should be put under close observation for at least one day in the hospital to ensure they do not develop unstable circulation or anemia requiring transfusion.

## Conclusions

We discussed the case of a patient who had moderate injuries with stable vital signs on arrival but developed cardiac arrest within six hours of the accident due to hemorrhaging and hyperkalemia. Trauma patients with markedly high FDP levels on arrival may develop hemorrhagic shock requiring massive transfusion and are at the risk of an impending hyperkalemic cardiac arrest. Hence, such trauma patients should be considered for admission to the hospital for observation, even if their initial vital signs are stable and their trauma mild or moderate.
